# Misinformation, Freedom of Expression and Mandatory Vaccination

**DOI:** 10.1093/phe/phag013

**Published:** 2026-07-13

**Authors:** Steven R Kraaijeveld

**Affiliations:** Department of Law, Ethics & Medical Humanities, Amsterdam UMC, Amsterdam, The Netherlands

## Abstract

In their recent book, *Inducing Immunity* (2024), Roland Pierik and Marcel Verweij develop an account of collective immunization according to which liberal-democratic governments are sometimes justified in restricting people's freedom to refuse vaccination. In this commentary, I focus on a specific argument in the book about misinformation, freedom of expression and mandatory vaccination. Pierik and Verweij argue that, when vaccine misinformation threatens vaccination rates, governments—in order to increase or maintain vaccination rates—should consider mandatory vaccination before restricting freedom of expression. I summarize the argument and raise two issues to help advance the debate.

## Introduction

In their recent book, *Inducing Immunity* (2024), Roland Pierik and Marcel Verweij offer a sustained and measured account of regulating collective immunization that involves setting limits to individual freedoms under certain epidemiological and social–political circumstances. The main argument of the book, in my understanding, can be summarized as follows. When vaccination rates fall below certain thresholds, the risk of disease outbreaks increases, which in turn risks harm to individuals and the collective good of protection against infectious diseases. Liberal-democratic governments have a responsibility to safeguard public health, which includes protecting citizens against the harms of infectious diseases and more generally preventing the social disruption that often accompanies epidemics. This responsibility entails that governments must act when vaccination rates drop below critical thresholds or in the face of dangerous disease outbreaks. Drawing on John Stuart Mill's liberal philosophy and, in particular, on the harm principle, the upshot of the arguments in the book is that there are moral and political grounds to justify mandatory vaccination.

The book also offers a defense of freedom of expression in the face of public criticism surrounding vaccination. Vaccine hesitancy can be amplified by the spread of online misinformation (Loomba *et al.*, 2021)—which, as Pierik and Verweij point out, raises the question of potentially suppressing vaccine-critical speech. However, the authors offer a strong argument for democratic states to respect freedom of expression, and thus for the need to tolerate at least some misinformation. The argument is rooted in a Millian defense of free speech and is partly grounded in the importance of government trustworthiness. The idea is that the overall success of immunization programs crucially depends on such programs being trustworthy to the public. Governments should do their best to be—and must *actually* be—trustworthy, instead of trying to manipulate trust in various ways (e.g. through explicit strategies aimed at psychological and behavioral change, like nudging). That is, the focus for public health professionals and authorities ‘should be on being trustworthy, not on inducing people to trust a vaccine’ (Pierik and Verweij, 2024: 178).

In their discussion of the importance of freedom of expression in relation to misinformation and vaccine hesitancy, Pierik and Verweij suggest that, in light of their previously summarized arguments, ‘[i]f partly due to misinformation, vaccine coverage is in decline and falls below a certain threshold minimum […], a liberal-democratic government better implements mandatory immunization’ (2024: 184).

To the best of my knowledge, the argument linking freedom of expression to mandatory vaccination is original to the book. Although it is discussed relatively briefly, it is worth addressing, as the argument and its consequences will be of interest to scholars as well as to the public. Before doing so, it is important to say a bit more about mandatory vaccination as it discussed in the book.

## The Goal of Mandatory Vaccination

The major purpose of vaccination, which the book underscores throughout, is to help protect societies against the harms of disease. This is why the harm principle is invoked in order to justify mandatory vaccination: if a substantial group of citizens do not get vaccinated, then this may either harm others directly^1^ through interpersonal infection, or indirectly through a failure to contribute to the collective good of group-level protection against infectious diseases.^2^ It is worth noting that, at least as far as the application of the harm principle goes, this is a consequentialist argument in nature. Citizens getting vaccinated is morally good because it prevents or reduces (the risk of) disease-caused harms and/or the need for freedom-restricting measures (as with disease outbreaks like COVID-19). An insufficient number of people getting vaccinated is morally bad because it increases (the risk of) disease-caused harms and/or the need for restrictive infectious disease measures. The moral badness of the harms that result from not getting vaccinated is what grounds the harm principle as a justification for restricting people's freedom—in this case, the freedom to decide for oneself whether or not to get vaccinated.

However, when misinformation is widespread, one wonders whether making vaccination mandatory will, in fact, advance the intended goal of increasing vaccine uptake and thus of reducing the harms associated with disease. And, given that misinformation about vaccines is often associated with a lack of trust in government and health programs as such (Jennings *et al.*, 2021), would the move to mandatory vaccination not risk further undermining trust and, accordingly, vaccination rates? Differently put: could mandatory vaccination, despite its moral justification through the harm principle, ultimately lead to (morally) worse outcomes? In what follows, I expand on these two issues and situate them within some of the book's larger aims.

## Will Mandatory Vaccination Increase Vaccine Uptake?

In cases where misinformation threatens to undermine vaccination rates, the extent to which making vaccination mandatory will affect vaccination rates is primarily an empirical question. While the book mentions some examples, like the case of Italy where the implementation of mandatory vaccination did not appear to have decreased vaccination rates, more systematic data are needed to understand the full and localized effects of such policies. While systematic reviews of the effects of mandatory vaccination often point toward short-term increases in uptake, causal effects are very difficult to determine and long-term effects are often unclear (e.g. Pitts *et al.*, 2014; Greyson *et al.*, 2019; Odone *et al.*, 2021). Importantly, the vast majority of studies on the effects of mandatory vaccination concern childhood vaccination or more circumscribed professional settings (e.g. mandatory vaccination for healthcare workers). Mandatory vaccination of entire adult populations is highly understudied, which is unsurprising given the relative historical scarcity of such far-reaching measures. While some studies of mandatory COVID-19 vaccination policies show an initial increase in uptake (e.g. Karaivanov *et al.*, 2022; Mello *et al.*, 2022), more robust and systematic evidence is needed to determine the broader effects of such policies.

It thus remains largely an open question whether mandatory vaccination of adults, for instance in cases of outbreaks of novel diseases like COVID-19, will reliably increase vaccination rates beyond a certain threshold—especially in light of a relatively new phenomenon like widespread online misinformation. Reactance against mandates—and, more generally, negative downstream effects—remain a concern. Some research suggests that, when it came to COVID-19 vaccine mandates, reactance ‘due to a mandate was positively associated with intentions to avoid […] COVID-19 vaccination and an unrelated chickenpox vaccination; it was [also] negatively associated with intentions to show protective behaviors limiting the spread of the coronavirus’ (Sprengholz *et al.*, 2021: 986). Of course, as Pierik and Verweij point out, the principle of proportionality requires that government interventions are suitable for achieving the stated purpose, which ‘can include a requirement that there is (scientific) evidence to show that the measure will [achieve its purpose]’ and not be counterproductive (2024: 55). The proportionality requirement might therefore be difficult to assess when evidence regarding the effects of mandatory vaccination for adult citizens on a large scale is limited.

The empirical matters of fact related to mandatory vaccination policies and their effects within a given setting will be key, including when weighed against the effects of misinformation. Minimal positive or long-term negative impacts of mandates on vaccine uptake, as well as reasonable uncertainty about the effects, will reduce the scope of the argument.

## How Does Mandatory Vaccination Relate to Trustworthiness?

Pierik and Verweij are keenly aware that trust is a hugely important and precarious good. It is one of the reasons why they argue, I think convincingly, against censoring free speech in response to misinformation: ‘we see this [suppression of information] as a wrong-headed approach to trying to maintain trust: a government that suppresses opinions, even those that are blatantly objectionable, is not trustworthy at all’ (2024: 180). If—partly due to misinformation—vaccine coverage falls below a certain threshold, the idea is that mandatory vaccination, although also constituting ‘a severe constraint of the freedom of citizens,’ nevertheless ‘fits much better in a trustworthy government policy’ (2024: 184). Seven well-considered requirements for trustworthy vaccination policies are laid out in the book, which I cannot do justice here, but which include requirements like transparency, a grounding in reasonable values and scientific evidence, and taking citizens’ concerns seriously.^3^

An issue that seems to remain, however, even if it is acknowledged in the book, is that coercion is notoriously difficult to align with trust. When it comes to vaccination, coercive measures can ultimately harm trust (Bester, 2015; Bardosh *et al.*, 2022). The risk is probably greatest when trust in governments and science is already at a low point; when vaccination is associated with high degrees of polarization (Rudolph, 2025) and moralization (Kraaijeveld and Jamrozik, 2022); and in cases where it concerns novel vaccines with less robust or established safety profiles (as with the COVID-19 vaccines). Yet, that is precisely when vaccine uptake is especially likely to be lower—and, therefore, when the need to increase uptake will be more pressing. Considering all of this, how does mandatory vaccination relate to trust and trustworthiness?

One of the proposed requirements in the book for trustworthiness is that vaccination should not simply be forced or imposed on citizens (criterion 5; p. 188). Room should be left to opt out: ‘[by] emphasizing that immunization is the right choice yet enabling citizens to make their own choice and, if they really want to, to opt out, the government is inviting them to trust the program’ (2024: 188). This seems to me to be rightly conducive to building trust (along with other requirements). A gray area does, however, open up: ‘if the threat of a possible outbreak is real, governments have reason to be less tolerant of citizens who refuse to participate’ (2024: 188). This inevitably raises the question of how stringent opt-outs ought to be in the face of outbreaks, while also meeting the fifth requirement of trustworthiness. Without the possibility of opting out of a mandate, the criterion of trustworthiness is unmet; while too much leniency defies the purpose of the mandate at some point along the spectrum of opt-out possibilities.

While this matter is not fully addressed and deserves more attention, it is a strength of the book that it allows for—rather than avoids—considerable nuance and complexity.

## Notes

1 It should be noted that, as Pierik and Verweij point out, the case of directly harming others through non-vaccination is not always straightforward; in cases where vaccines do not provide sterilizing immunity or are associated only with small reductions in transmission and/or other viable means of reducing the spread of disease, the harm principle may not directly apply as a justification for mandatory vaccination (cf. Kraaijeveld, 2023a, 2023b; Kraaijeveld et al., 2024).

2 In this commentary, I focus on mandatory vaccination for adults.

3 For a full discussion of the seven requirements, see pages 185–189.
